# An interpretable prediction model for non-invasive identification of the phlegm-dampness constitution based on machine learning

**DOI:** 10.3389/fendo.2026.1929926

**Published:** 2026-09-22

**Authors:** Xi Zhang, Tianxing Li, Yajie Liu, Liujie Zhu, Yunlong Yao, Yunzhi Zhang, Chenyu Fei, Shuo Huang, Zhihui Xu, Fei Ye, Yifan Xia, Lingru Li

**Affiliations:** 1 Wangqi Academy, National Institute of TCM Constitution and Preventive Medicine, Beijing University of Chinese Medicine, Beijing, China; 2 Zhongshan City People’s Hospital, Zhongshan, China; 3 Otolaryngology Department of Huangpu People’s Hospital of Zhongshan, Zhongshan, China

**Keywords:** constitution of traditional chinese medicine, machine learning, metabolic diseases, phlegm-dampness constitution, shapley additive explanations

## Abstract

**Introduction:**

In traditional Chinese medicine (TCM), constitutional types influence disease susceptibility. Phlegm-Dampness Constitution (PDC), a common subtype, has been linked to elevated risk of metabolic diseases. Therefore, early identification of PDC is essential for implementing early interventions and disease prevention strategies. This study aimed to develop and evaluate interpretable machine learning models for the identification of PDC based on routinely available clinical variables and dietary preferences.

**Methods:**

We analyzed 139 participants (96 PDC and 43 Balanced Constitution [BC]). Seventeen clinical and biochemical variables were screened using least absolute shrinkage and selection operator regression, and logistic regression, random forest, support vector machine, and Extreme Gradient Boosting classifiers were developed. The dataset was divided into training and test sets, and three-fold cross-validation was applied. Model performance was assessed using the area under the receiver operating characteristic curve (AUC), cut-off value, sensitivity, specificity, positive likelihood ratio (PLR), negative likelihood ratio (NLR), positive predictive value (PPV), and negative predictive value (NPV), while model outputs were interpreted using SHapley Additive exPlanations (SHAP).

**Results:**

The logistic regression model using waist circumference and triglycerides (TG), with age and body mass index (BMI) as covariates, achieved an AUC of 0.983, with a sensitivity of 0.945 and a specificity of 0.970. A second fully non-invasive model using systolic blood pressure, diastolic blood pressure, and waist circumference, with the same covariates, also showed good discrimination and was retained as a candidate model. SHAP analysis provided individual-level explanations of model predictions and identified BMI and TG as key clinical contributors. In addition, we constructed a PDC identification model based on dietary preferences. The logistic regression model showed good discriminative performance in the training data (AUC = 0.891, specificity = 0.964) and the testing data (AUC = 0.838, specificity = 0.894). SHAP indicated that scorching and salty preferences shifted predictions toward PDC, whereas a light preference shifted predictions toward BC.

**Discussion:**

In summary, this study developed interpretable models to improve PDC identification, including a fully non-invasive model, thereby potentially supporting early risk identification and preventive strategies for metabolic diseases in clinical settings.

## Introduction

The growing prevalence of metabolic diseases is likely to adversely impact the quality of life for nearly two billion people globally (1). Such diseases, including obesity, type 2 diabetes (T2D), hyperlipidemia, and metabolic syndrome among others (2), significantly contribute to global mortality rates. Moreover, most of them present with a long, insidious course (3), with their early symptoms, such as a slight increase in weight, blood glucose, or lipid levels, often easily overlooked (3–5). Therefore, it is imperative to develop early detection and preventive measures to reduce the magnitude of metabolic disease burden on individuals, particularly in high-risk populations (6).

In traditional Chinese medicine (TCM), the constitutional theory classifies individuals based on their unique physiological and pathological characteristics. One such type is the phlegm-dampness constitution (PDC), which affects approximately 7.32% of the Chinese population (7). Individuals with PDC typically present with symptoms such as a sensation of heaviness, chest tightness, abdominal distension, a greasy forehead, eyelid swelling, and a thick tongue coating (8). Studies have shown that this constitution increases the risk of metabolic diseases (9), and individuals with PDC are more prone to developing conditions such as obesity, T2D, and hypertriglyceridemia (10). In contrast, individuals with a balanced constitution (BC) are regarded as embodying optimal health in TCM. They typically exhibit high energy levels, a resilient immune system, a steady and robust pulse, and the ability to achieve restorative sleep (8).

Although conventional approaches for assessing constitution, including TCM constitution scales, aim for objectivity, they can be influenced by subjective factors and convoluted scoring systems and calculation methods. This complexity can hinder the accurate classification of constitutions and reduce the effectiveness of identifying high-risk groups, such as individuals with PDC. Therefore, a rapid, objective, and clinically accessible screening approach that minimizes reliance on subjective assessment and invasive laboratory testing is needed.

Recent machine learning (ML) studies have applied data-driven methods to TCM constitution identification. Sun et al. developed a questionnaire-based ML approach for the rapid identification of multiple TCM constitution types (11), whereas Li et al. constructed an interpretable model based on genetic indicators for Yin-deficiency constitution (12). For PDC specifically, previous models have used metabolic and immune laboratory parameters (13) or three-dimensional body-shape features (14). Deep learning models have also been developed for the classification of nine TCM constitution types (15). However, these approaches have generally focused on multi-constitution classification or relied on laboratory, genetic, or specialized imaging features, which may limit their accessibility in primary care and community-based screening. The use of both linear and nonlinear algorithms allows different patterns of association with constitution status to be evaluated, while interpretable approaches such as SHapley Additive exPlanations (SHAP) can further clarify the contribution of individual features to model predictions. Moreover, routinely available anthropometric and blood-pressure measurements and dietary-preference information have not been sufficiently evaluated within a PDC-specific and interpretable framework. Differences in dietary preferences may reflect both metabolic health and TCM constitution, as taste alterations have been reported in adults with obesity (16). Dietary habits, including a light diet and the consumption of fatty, sweet, salty, barbecued, and pungent foods, have also been associated with phlegm-wetness constitution (17). We therefore evaluated self-reported dietary preferences as accessible, non-invasive candidate features. To address these gaps, we compared logistic regression, random forest, support vector machine, and XGBoost for the identification of PDC, with BC serving as the reference group. We also used SHAP to examine overall feature importance and individual predictions. By evaluating these complementary clinical and dietary approaches within the same cohort, this study provides a practical and interpretable framework for PDC identification.

## Methods

### Ethical approval

The research protocols complied with all relevant ethical regulations and were approved by the Institutional Review Board and Ethics Committee of Beijing University of Chinese Medicine (2017BZHYLL0406).

### Participants

Participants aged 18–50 residing in Beijing were recruited. Following TCM constitution identification, 139 participants were included in the analysis, comprising 96 cases of PDC and 43 cases of BC. The classification criteria for PDC and BC were established according to the 2009 Standards for Classification and Judgment of TCM Constitution (18). A flowchart for the inclusion and exclusion criteria is displayed in **Figure 1**.

**Figure 1:**
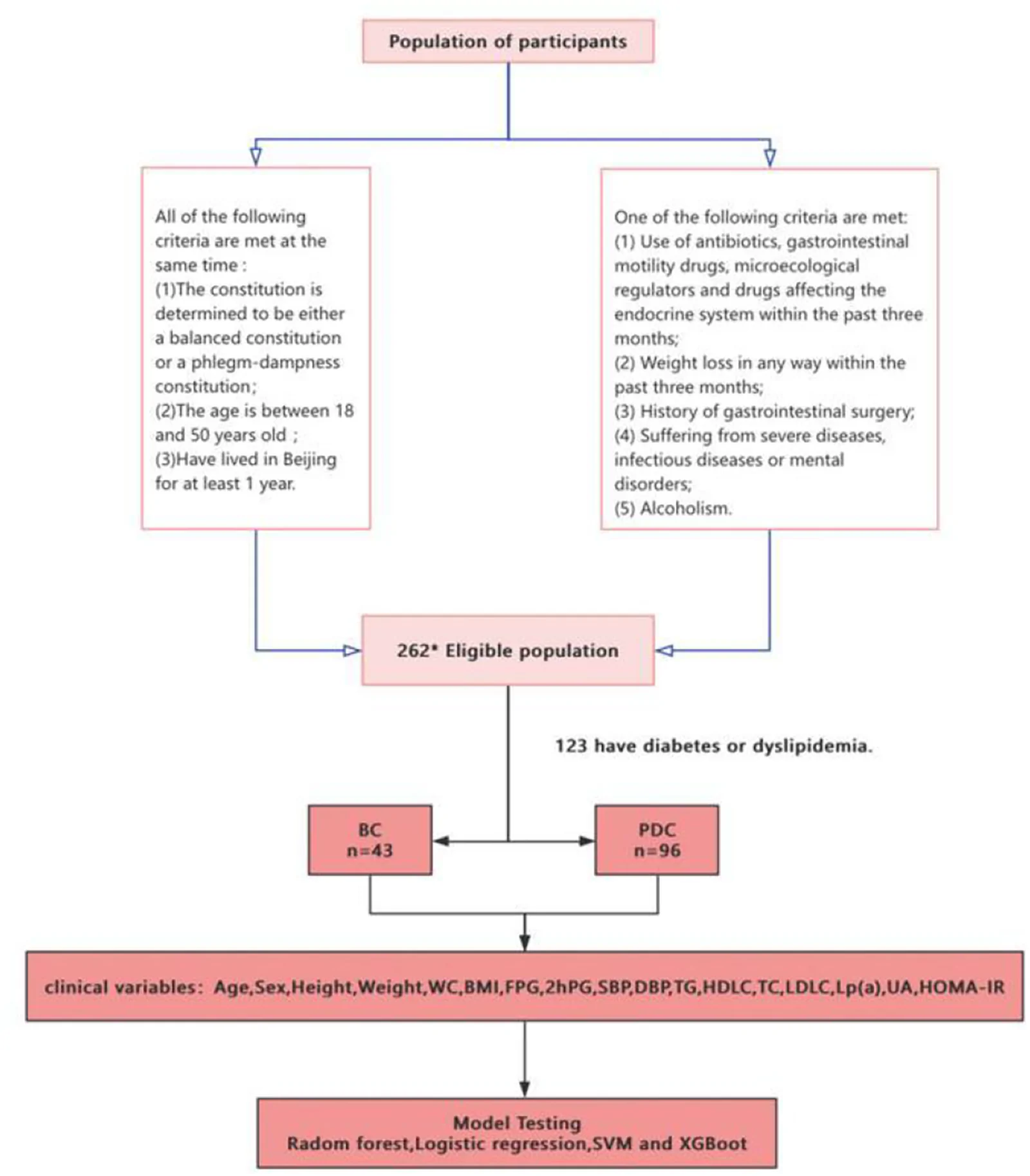
Flowchart of inclusion and exclusion criteria.

Inclusion criteria: (1) Participants who fulfill the requirements for the BC/PDC constitution (ZYXH/T157-2009); (2) aged between 18 and 50 years; and (3) resided in Beijing for a minimum of one year.

Exclusion criteria: (1) Use of antibiotics, gastrointestinal stimulants, microecological regulators, or endocrine-affecting drugs in the last three months; (2) weight loss via any pharmacological methods in the past three months; (3) a history of gastrointestinal surgery; (4) diagnosis of a serious illness, infectious disease, or mental disorder; and (5) a history of alcoholism.

### Data collection

Questionnaire data, including age (years) and sex, were collected through personal interviews conducted by trained clinical staff. The same clinical staff performed anthropometric and blood-pressure measurements, including height (m), weight (kg), waist circumference (WC, cm), systolic blood pressure (SBP, mmHg), and diastolic blood pressure (DBP, mmHg), according to standardized procedures. Body mass index (BMI, kg/m²) was calculated from height and weight. Hematology and biochemical analysis were conducted by qualified laboratory personnel on blood samples obtained after an overnight fasting period. After analysis, biochemical data were obtained, including fasting blood glucose (FBG, mmol/L), 2-h postprandial glucose (2hPG, mmol/L), triglyceride (TG, mmol/L), high-density lipoprotein cholesterol (HDLC, mmol/L), total cholesterol (TC, mmol/L), low-density lipoprotein cholesterol (LDLC, mmol/L), lipoprotein(a) (Lp(a), mg/dL), uric acid (UA, μmol/L), and homeostasis model assessment-insulin resistance (HOMA-IR). Participants with abnormalities of glycolipid metabolism were excluded based on the following diagnostic criteria: dyslipidemia was defined as having at least one of the following: fasting TC ≥5.2 mmol/L, TG ≥1.7 mmol/L, LDL-C ≥3.4 mmol/L, or HDL-C <1.0 mmol/L (19), and abnormal blood glucose as indicated by an FBG >5.6 mmol/L (20).

### Data preprocessing

The outcome variable was binary TCM constitution status, defined as PDC versus BC according to ZYXH/T157-2009, with BC serving as the reference category. No missing values were identified in any of the variables included in the analysis; therefore, no participants were excluded because of missing data, and no imputation was required. Before model development, continuous variables underwent data-quality checks for physiologically or clinically implausible values. When an apparent recording error could be verified against the source records, the value was corrected or recalculated from the original measurements where possible. Outliers were subsequently screened using the interquartile range (IQR) method. Observations identified as statistical outliers but remaining physiologically or clinically plausible after verification were retained.

### Dataset partitioning

The dataset was randomly divided into training and test sets at a ratio of 7:3 using stratified sampling according to PDC/BC status to preserve the relative distribution of PDC and BC participants in the training and test sets. To account for differences in measurement scales among continuous predictors, Z-score standardization was applied before model fitting. The mean and standard deviation used for standardization were estimated from the training set only, and the same transformation parameters were subsequently applied to the test set. Three-fold cross-validation was performed for model development.

### Feature selection and model development

Least absolute shrinkage and selection operator (LASSO) regression was performed in R version 4.0.3 using the glmnet package for feature selection (21). The regularization parameter was selected using three-fold cross-validation based on the minimum cross-validation error criterion (lambda.min), and variables with non-zero coefficients at lambda.min were retained for subsequent model development. Four machine-learning classifiers were then developed in Python version 3.11.6 for PDC prediction: logistic regression, random forest, support vector machine (SVM), and Extreme Gradient Boosting (XGBoost). Logistic regression, random forest, and SVM were implemented using scikit-learn (version 1.4.2), whereas XGBoost was implemented using the XGBoost package (version 1.7.5).

### Hyperparameter tuning

Hyperparameter tuning was conducted exclusively using three-fold cross-validation, with the mean cross-validated area under the receiver operating characteristic curve (AUC) used as the criterion for parameter selection. For the random forest model, mtry and ntree were optimized; for SVM with a radial basis function kernel, the cost parameter (C) and γ were tuned; and for XGBoost, eta, max_depth, subsample, and colsample_bytree were optimized. The candidate parameter ranges are summarized in **Supplementary Table S1**.

### Model performance evaluation and interpretation

The performance of the model was assessed using the receiver operating characteristic (ROC) curve generated by the R package pROC 1.18.0, along with its AUC. The cut-off value, diagnostic sensitivity, specificity, positive likelihood ratio (PLR), negative likelihood ratio (NLR), positive predictive value (PPV), negative predictive value (NPV) of the model were determined. For pairwise model comparisons, AUCs calculated on the independent test set were compared using the DeLong test, facilitating a comparative analysis of their predictive abilities. Decision curve analysis (DCA) was performed in the training and test sets using the dcurves package (version 1.1.0). SHAP analysis was performed using the shap package (version 0.43.0) with the TreeSHAP algorithm. Both analyses were implemented in Python 3.11.6.

### Statistical analysis

Statistical analyses were performed using IBM SPSS Statistics version 29.0. All statistical tests were two-sided, with a significance level of *P* < 0.05. The distribution of continuous variables was assessed using the Shapiro-Wilk test and quantile-quantile (Q-Q) plots. Continuous variables were summarized as mean ± standard deviation (SD). Normally distributed continuous variables were compared using the independent-samples t-test, whereas non-normally distributed continuous variables were compared using the Wilcoxon rank-sum test. Categorical variables were presented as counts and compared using the chi-square test.

## Results

### Comparison of clinical characteristics

The clinical characteristics of the study participants are summarized in **Table 1**. The mean age was 32.51 ± 8.334 for the PDC group and 27.33 ± 6.290 for the BC group. The two groups exhibited statistically significant age differences (*P* < 0.05), with the PDC group being older than the BC group. Significant differences were also found in weight, WC, BMI, TG, TC, and HOMA-IR between the groups (*P* < 0.05). However, no statistically significant differences were observed in sex, height, FBG, 2hPG, SBP, DBP, LDLC, HDLC, Lp(a), and UA (*P* > 0.05).

**Table 1:** Clinical characteristics of PDC and BC groups (*P < 0.05).

Characteristic	BC group(n=43)	PDC group(n=96)	*P*
Age (years)	27.330 ± 6.290	32.510 ± 8.334	<0.01^*^
Sex (Female/Male)	28/15	52/44	N/A
Height (m)	1.669 ± 0.0750	1.677 ± 0.0832	N/A
Weight (kg)	59.793 ± 7.549	77.993 ± 16.542	0.002^*^
WC (cm)	75.791 ± 5.998	94.599 ± 11.468	0.004*
BMI (kg/m²)	21.274 ± 1.690	27.347 ± 4.155	<0.001*
FBG (mmol/L)	4.531 ± 0.435	5.143 ± 1.022	N/A
2hPG (mmol/L)	4.789 ± 1.010	5.144 ± 1.022	N/A
SBP (mmHg)	111.700 ± 10.315	118.300 ± 12.875	N/A
DBP (mmHg)	72.330 ± 7.849	78.730 ± 8.759	N/A
TG (mmol/L)	0.677 ± 0.235	0.993 ± 0.301	0.021*
HDLC (mmol/L)	1.385 ± 0.233	1.200 ± 0.227	N/A
TC (mmol/L)	4.198 ± 0.654	4.336 ± 0.484	0.033*
LDLC (mmol/L)	2.122 ± 0.548	2.463 ± 0.442	N/A
Lp(a) (mg/dL)	11.516 ± 12.663	10.560 ± 12.076	N/A
UA (μmol/L)	292.279 ± 79.888	334.467 ± 75.671	N/A
HOMA-IR	1.837 ± 0.612	2.893 ± 1.338	<0.001*

### Screening of clinical variables for PDC

LASSO regression analysis was conducted utilizing 17 variables (**Figure 2**). Independent predictive indicators were identified in the training set based on nonzero coefficients obtained from the LASSO regression. Subsequently, the optimal parameter lambda was determined through threefold cross-validation. The results showed that four variables remained significant, namely age, WC, BMI, and TG, at a lambda value of 0.109. These four variables were therefore used as the main candidate predictors in the subsequent model development.

**Figure 2:**
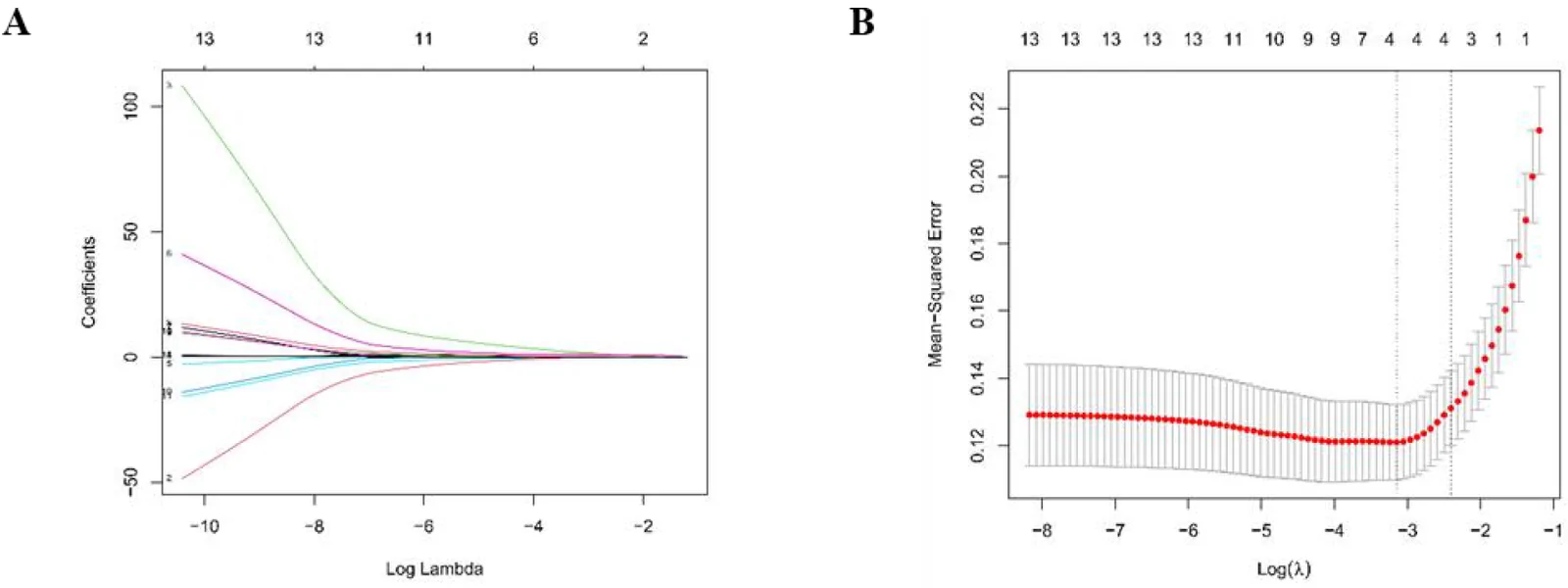
LASSO regression analysis for predictor selection. **(A)** Coefficient profiles of the candidate variables as a function of log(λ). **(B)** Cross-validation was utilized to generate vertical lines at the selected values.

### Comprehensive analysis of prediction models with multiple indicators

Multi-variable prediction models for PDC identification were constructed using four ML methods: logistic regression, random forest, SVM, and XGBoost. For each ML method, we constructed 6 multi-variable prediction models (M1-M6) incorporating various combinations of 12 indicators (age, BMI, WC, TG, TC, HDLC, LDLC, SBP, DBP, FBG, 2hPG, and UA), identified through multiple statistical analysis, LASSO-based variable selection results, and existing literature review, which confirmed their strong associations with PDC (17, 18). The establishment of model groupings were defined as follows: M1 included WC and TG; M2 comprised SBP, DBP, and WC; M3 encompassed TC, TG, HDLC, LDLC, FBG, 2hPG, and UA. Furthermore, M4 contained SBP, DBP, WC, TC, TG, HDLC, LDLC, FBG, 2hPG, and UA. In contrast, M5 contained WC, while M6 contained TG. In all models, age and BMI were included as covariates to analyze the real-world data (M1-M6). The performance of the prediction models was evaluated using the AUC values. In terms of their intended roles, M1 combined central adiposity and lipid-related information, M2 represented a fully non-invasive anthropometric and hemodynamic feature set, M3 and M4 evaluated broader biochemical or combined panels, and M5 and M6 served as parsimonious benchmark models.

**Figure 3** shows the ROC curves of all predictive models using combined indicators. The highest AUC values for logistic regression, random forest, SVM, and XGBoost were 0.983, 0.972, 0.983, and 0.965, respectively. Sensitivity, specificity, PLR, NLR, PPV, NPV, and their corresponding 95% CIs are reported in **Table 2**.

**Figure 3:**
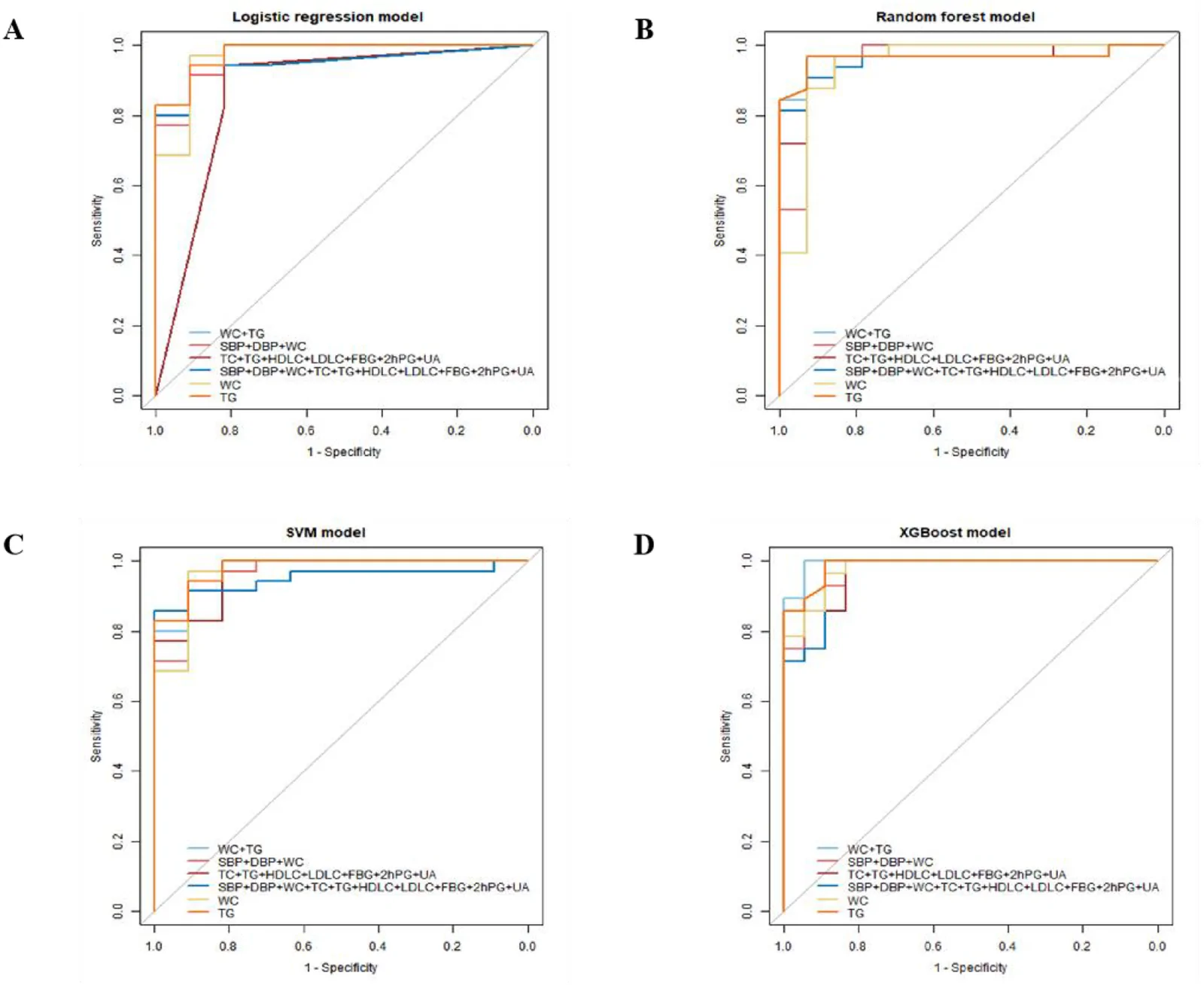
ROC curves for prediction models utilizing four ML methods. **(A)** Logistic regression. **(B)** Random forest. **(C)** SVM. **(D)** XGBoost.

**Table 2:** M1–M6 performance metrics for distinguishing PDC from BC participants using logistic regression, random forest, SVM, and XGBoost methods.

Performance metric	M1	M2	M3	M4	M5	M6
Logistic regression						
AUC	0.983	0.977	0.914	0.875	0.977	0.982
95% CI	0.953-1.000	0.942-1.000	0.822-0.999	0.788-0.958	0.938-1.000	0.951-1.000
Cut-off	0.628	0.681	0.534	0.500	0.484	0.570
Sensitivity	0.945	0.919	0.905	0.851	0.980	0.937
95% CI	0.868-1.000	0.828-0.997	0.804-0.997	0.731-0.963	0.942-1.000	0.852-1.000
Specificity	0.970	0.933	0.884	0.859	0.890	0.951
95% CI	0.913-1.000	0.828-1.000	0.750-1.000	0.729-0.961	0.731-1.000	0.859-1.000
PLR	31.500	13.716	7.802	6.035	8.909	19.122
95% CI	5.753-172.479	4.489-41.911	3.410-17.850	2.872-12.681	3.805-20.858	5.120-71.426
NLR	0.057	0.087	0.107	0.173	0.022	0.066
95% CI	0.015-0.247	0.028-0.288	0.036-0.321	0.080-0.429	0.003-0.219	0.017-0.255
PPV	0.990	0.967	0.945	0.921	0.948	0.978
95% CI	0.942-0.998	0.908-0.989	0.878-0.976	0.845-0.961	0.885-0.977	0.924-0.994
NPV	0.900	0.829	0.810	0.735	0.946	0.868
95% CI	0.782-0.958	0.699-0.910	0.676-0.897	0.601-0.836	0.830-0.984	0.743-0.937
Random forest						
AUC	0.972	0.955	0.950	0.948	0.954	0.954
95% CI	0.939-1.000	0.895-1.000	0.889-1.000	0.887-1.000	0.888-1.000	0.897-1.000
Cut-off	0.549	0.585	0.513	0.543	0.534	0.581
Sensitivity	0.938	0.937	0.979	0.958	0.927	0.948
95% CI	0.875-0.990	0.870-1.000	0.939-1.000	0.904-1.000	0.845-0.990	0.874-1.000
Specificity	0.929	0.929	0.905	0.905	0.929	0.905
95% CI	0.857-1.000	0.823-1.000	0.788-1.000	0.788-1.000	0.823-1.000	0.788-1.000
PLR	13.211	13.197	10.305	10.084	13.056	9.979
95% CI	4.476-38.997	4.471-38.956	4.095-25.937	4.005-25.393	4.422-38.548	3.962-25.134
NLR	0.067	0.068	0.023	0.046	0.079	0.057
95% CI	0.025-0.279	0.023-0.328	0.004-0.279	0.013-0.279	0.028-0.283	0.016-0.270
PPV	0.971	0.967	0.960	0.960	0.968	0.958
95% CI	0.914-0.991	0.908-0.989	0.901-0.984	0.900-0.985	0.909-0.989	0.897-0.984
NPV	0.885	0.871	0.949	0.910	0.861	0.884
95% CI	0.762-0.949	0.745-0.940	0.835-0.986	0.788-0.965	0.735-0.933	0.757-0.949
SVM						
AUC	0.983	0.970	0.938	0.945	0.975	0.983
95% CI	0.954-1.000	0.927-1.000	0.871-0.996	0.879-1.000	0.935-1.000	0.955-1.000
Cut-off	0.521	0.567	0.544	0.631	0.506	0.569
Sensitivity	0.970	0.920	0.900	0.892	0.970	0.937
95% CI	0.925-1.000	0.864-0.990	0.816-0.984	0.809-0.974	0.925-1.000	0.852-1.000
Specificity	0.933	0.914	0.902	0.944	0.890	0.951
95% CI	0.828-1.000	0.800-1.000	0.778-1.000	0.887-1.000	0.731-1.000	0.859-1.000
PLR	14.478	10.698	9.184	15.929	8.818	19.122
95% CI	4.743-44.192	4.030-28.395	3.699-22.798	4.660-54.450	3.766-20.651	5.120-71.426
NLR	0.032	0.088	0.111	0.114	0.034	0.066
95% CI	0.007-0.232	0.028-0.340	0.046-0.304	0.050-0.375	0.007-0.252	0.017-0.255
PPV	0.968	0.958	0.955	0.968	0.948	0.978
95% CI	0.911-0.989	0.895-0.984	0.891-0.982	0.907-0.989	0.885-0.977	0.924-0.994
NPV	0.928	0.857	0.802	0.796	0.925	0.868
95% CI	0.811-0.975	0.730-0.930	0.669-0.890	0.667-0.884	0.804-0.974	0.743-0.937
XGBoost						
AUC	0.961	0.965	0.954	0.960	0.960	0.956
95% CI	0.899-1.000	0.904-1.000	0.879-1.000	0.890-1.000	0.896-1.000	0.885-1.000
Cut-off	0.553	0.539	0.328	0.491	0.386	0.654
Sensitivity	0.970	0.934	0.980	0.968	0.968	0.941
95% CI	0.925-1.000	0.851-1.000	0.942-1.000	0.918-1.000	0.908-1.000	0.878-0.997
Specificity	0.927	0.927	0.890	0.933	0.885	0.933
95% CI	0.791-1.000	0.791-1.000	0.731-1.000	0.828-1.000	0.719-1.000	0.828-1.000
PLR	13.288	12.795	8.909	14.448	8.417	14.045
95% CI	4.577-38.573	4.404-37.169	3.805-20.858	4.733-44.103	3.671-19.303	4.599-42.895
NLR	0.032	0.071	0.022	0.034	0.036	0.063
95% CI	0.007-0.219	0.020-0.268	0.003-0.226	0.007-0.232	0.005-0.247	0.021-0.245
PPV	0.969	0.967	0.948	0.968	0.948	0.968
95% CI	0.913-0.989	0.908-0.989	0.885-0.977	0.911-0.989	0.885-0.977	0.910-0.989
NPV	0.925	0.875	0.946	0.939	0.924	0.870
95% CI	0.807-0.973	0.750-0.942	0.830-0.984	0.826-0.980	0.802-0.973	0.744-0.939

M1: “WC+TG”, M2: “SBP+DBP+WC”, M3: “TC+TG+HDLC+LDLC+FBG+2hPG+UA”, M4: “SBP+DBP+WC+ TC+TG+HDLC+LDLC+FBG+2hPG+UA”, M5: “WC”, M6: “TG”, and age and BMI covariates were added to M1-M6.

The AUC values for all models in logistic regression were only one unit less than 0.900. The highest AUC of models in logistic regression was 0.983 (M1: 95% CI: 0.953-1.000), with a cut-off value of 0.628, a sensitivity of 0.945 (95% CI: 0.868-1.000), and a specificity of 0.970 (95% CI: 0.913-1.000). Moreover, the PPV and NPV value of M1 was 0.990 and 0.900, supporting its overall predictive performance. Collectively, these findings indicated that M1 developed through logistic regression achieved the best prediction performance.

Five random forest models achieved AUC values ≥ 0.950. Notably, the highest AUC and specificity were 0.972 (M1: 95% CI: 0.939-1.000) and 0.929 (M1: 95% CI: 0.857-1.000), with a sensitivity of 0.938 (M1: 95% CI: 0.875-0.990), a PPV of 0.971 (M1: 95% CI: 0.914-0.991) and a NPV of 0.885 (M1: 95% CI: 0.762-0.949).

Among the SVM models, M6, which exclusively used TG as the primary predictor, achieved the high AUC of 0.983 (95% CI: 0.955-1.000). It exhibited a cut-off value of 0.569, with a sensitivity of 0.937 (95% CI: 0.852-1.000), a specificity of 0.951 (95% CI: 0.859-1.000), and a PPV of 0.978 (95% CI: 0.924-0.994). Compared with other ML models, SVM models displayed narrower confidence intervals, suggesting greater stability in their performance.

Among the XGBoost models, the highest AUC was 0.965 (M2: 95% CI: 0.904–1.000), which did not exceed the highest AUCs achieved by the other three ML methods. Given that this ML model consistently falls short in all critical data metrics, its reliability and effectiveness in practical applications remain questionable. Consequently, it lacks the advantages offered by the other models.

In summary, SVM M6 with one indicator (TG) and logistic regression M1 with two indicators (WC and TG) had the highest AUC (both 0.983). Both M1 and M6 models necessitate TG measurement, which requires blood testing. In addition, M1 mandates an extra measurement of WC. Regarding PPV and NPV, logistic regression M1 outperformed SVM M6, indicating that the former provides a more reliable foundation. This implies that logistic regression M1 has greater credibility.

### Hypothesis testing results in model comparison

Based on the aforementioned results, we selected and compared M1 model with five other suboptimal models in all ML methods and then subjected to hypothesis testing (**Table 3**). No significant difference was found between the optimal and prediction suboptimal models in logistic regression, random forest, SVM, and XGBoost, indicating that the optimal model does not offer a substantial advantage. Notably, the M2 model only included three non-invasive indicators: SBP, DBP, and WC, which warrant further attention. In the assessment of constitution, there was no significant difference between M1 and M2 (*P* > 0.05). Considering its efficiency, cost-effectiveness, and non-invasive nature, M2 may represent a practical and scalable candidate model for PDC.

**Table 3:** Comparative analysis of the AUC in predictive models.

	Index	M1 vs M2	M1 vs M3	M1 vs M4	M1 vs M5	M1 vs M6
Logistic Regression	AUC	0.979/0.971	0.979/0.870	0.979/0.951	0.979/0.969	0.979/0.979
	*P* value	0.330	0.060	0.360	0.391	1.000
	Index	M1 vs M2	M1 vs M3	M1 vs M4	M1 vs M5	M1 vs M6
Random Forest	AUC	0.973/0.955	0.973/0.960	0.973/0.973	0.973/0.944	0.973/0.965
	*P* value	0.460	0.539	1.000	0.371	0.735
	Index	M1 vs M2	M1 vs M3	M1 vs M4	M1 vs M5	M1 vs M6
SVM	AUC	0.940/0.937	0.940/0.927	0.940/0.923	0.940/0.900	0.940/0.897
	*P* value	0.591	0.623	0.144	0.677	0.604
	Index	M1 vs M2	M1 vs M3	M1 vs M4	M1 vs M5	M1 vs M6
XGBoost	AUC	0.979/0.966	0.979/0.964	0.979/0.951	0.979/0.969	0.979/0.979
	*P* value	0.240	0.290	0.360	0.771	1.000

### Identification of important features based on ML

In the process of evaluating the most effective predictive model, our objective is to identify key variables for predicting PDC to improve research accuracy. Nonlinear techniques such as random forest or XGBoost were employed to determine feature significance (**Figures 4A, B**). The results were compared with independent predictive features from LASSO regression, considering the common metrics across the three ML methods as the most significant for PDC. The findings were visually represented in a Venn diagram (**Figure 4C**). Key features selected included BMI, WC, and TG, with BMI and WC being non-invasive indicators. The recurrence of these variables across LASSO, random forest, and XGBoost suggests that anthropometric and lipid-related information contributed consistently to PDC identification in this cohort. The non-invasive candidate model, M2, also included BMI and WC, further supporting the relevance of these variables to non-invasive PDC identification.

**Figure 4:**
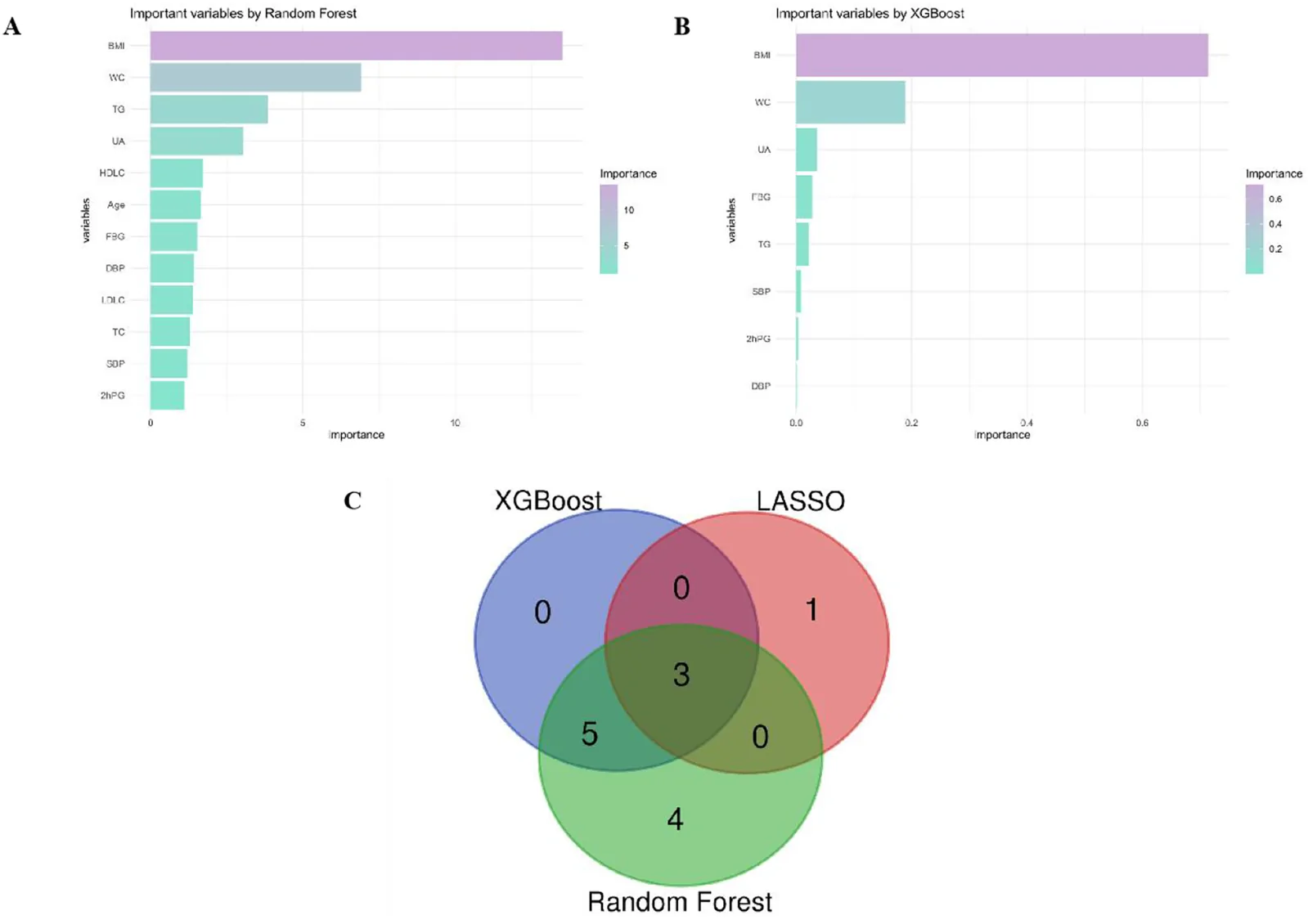
Identification of PDC features. **(A)** Variable significance selection using the random forest method. **(B)** Key feature selection using the XGBoost method. **(C)** Venn diagram showing the features common to LASSO, random forest, and XGBoost.

### Model interpretation using SHAP

SHAP was utilized to assess the importance of predictors in identifying PDC. A SHAP summary plot was generated to determine the most important predictors in developing the prediction model (**Figure 5A**). The SHAP feature importance bar plot provides a visual representation of the contribution levels of each feature (**Figure 5B**). BMI, TG, FBG, and SBP were the leading contributors to the clinical-model output. Clinically, these variables represent adiposity, lipid metabolism, glycemic regulation, and blood-pressure status, indicating that the model identified PDC primarily through a combination of anthropometric and metabolic characteristics. The inclusion of BMI and SBP also highlights the potential value of routinely obtainable clinical measurements for non-invasive PDC identification.

**Figure 5:**
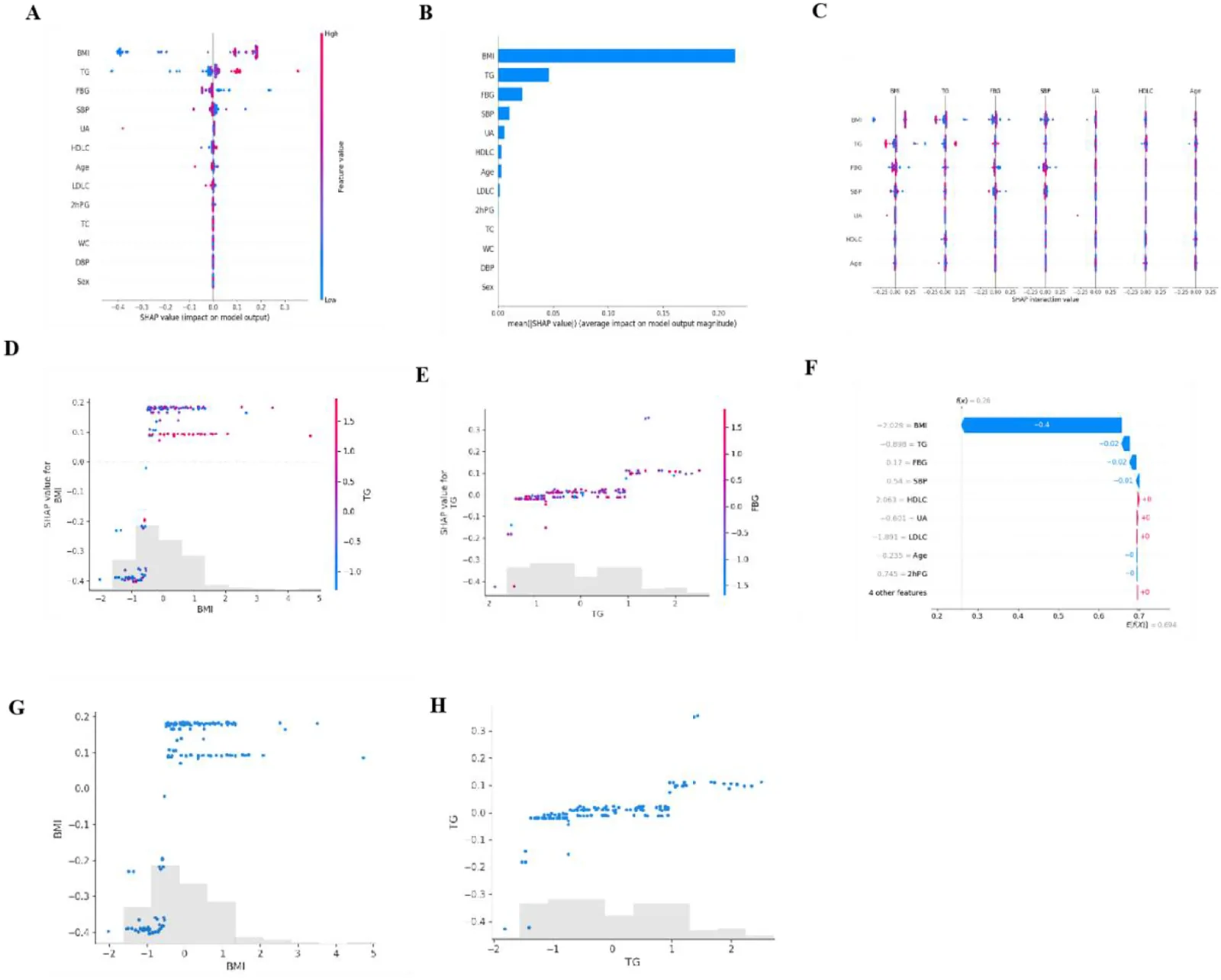
Model interpretation using SHAP. **(A)** SHAP summary plot displaying feature attributes. Each point represented an individual sample, with red indicating higher feature values and blue indicating lower feature values. **(B)** Bar plot indicating feature significance ranking according to SHAP. **(C)** SHAP interaction values. **(D)** SHAP interaction values between BMI and TG. **(E)** SHAP interaction values between TG and FBG. **(F)** Waterfall plot showing features as determined by SHAP. **(G)** Dependence plots for SHAP values related to BMI. **(H)** Dependence plots for SHAP values related to TG.

The present study utilized SHAP interaction value plots to explore the relationships among the top seven key features associated with metabolic diseases and PDC identification (**Figure 5C**). Through the identification of significant main and interaction effects, it was found that BMI, TG, FBG, and SBP had a substantial positive impact on PDC diagnosis. Higher values of these anthropometric and metabolic indicators generally produced stronger positive SHAP contributions, indicating a higher model-predicted probability of PDC when adiposity and metabolic abnormalities occurred together. Interactive visualizations of key indicators such as BMI, TG, and FBG are presented in **Figures 5D, E**. For instance, in the first image in **Figure 5D**, the x-axis denotes BMI values after robust scaling, while the y-axis shows SHAP interaction values between BMI and TG, illustrating how the interaction between BMI and TG contributed to the model output across samples. Additionally, we generated a waterfall plot for a randomly selected sample, which further validated the importance of the four indicators in identifying PDC (**Figure 5F**), thereby illustrating the clinical factors underlying an individual prediction.

The SHAP dependence plots were used to visualize the effects of individual features on the model output. Lower BMI and TG values were associated with negative SHAP values and shifted the model output toward BC, whereas higher values were associated with positive SHAP values and shifted the output toward PDC (**Figures 5G, H**). This directional pattern was consistent with the significantly higher BMI and TG levels observed in the PDC group, supporting the clinical relevance of adiposity and lipid-metabolism indicators for PDC identification. From the TCM perspective, the prominence of BMI- and adiposity-related features is consistent with the commonly described characteristics of PDC, including bodily heaviness and a tendency toward obesity (8, 9). Overall, the SHAP results highlighted BMI and TG as key contributors to PDC identification, while FBG and SBP provided complementary metabolic and clinical information. Notably, BMI and SBP are routinely obtainable clinical measures that may support non-invasive PDC identification. These findings further support the potential of clinically accessible variables for PDC screening.

### Prediction models for PDC based on dietary preferences

A non-invasive predictive model incorporating dietary preferences, including sweet, spicy, sour, salty, light, greasy, scorching, cold, hot, and tea, was constructed to enable swift and convenient predictions for PDC. Analysis of the ROC curves for the training dataset revealed that the XGBoost model achieved superior performance, with an AUC of 0.919 (95% CI: 0.867-0.972) (**Figure 6A**). The corresponding specificity was 0.969. The logistic regression and SVM models demonstrated strong performance, with the AUCs of 0.891 (95% CI: 0.828-0.953) and 0.884 (95% CI: 0.816-0.951), and specificity of 0.964 and 0.908, respectively. In contrast, the random forest model had a lower AUC of 0.796 (95% CI: 0.706-0.886), with a specificity value of 0.883, which indicated that their performance was weaker in the training data. In the test dataset, logistic regression achieved the highest AUC of 0.838 (95% CI: 0.721-0.954) (**Figure 6B**). The corresponding specificity was 0.894. In contrast, the performance of the XGBoost model on the test set was lower, yielding an AUC value of 0.733 (95% CI: 0.587-0.879), and specificity of 0.843. The random forest and SVM models demonstrated comparable performance, with AUC values of 0.775 (95% CI: 0.635-0.914) and 0.784 (95% CI: 0.649-0.919), respectively. Moreover, the specificity values were 0.835 and 0.850, respectively, further supporting their similar efficacy. In summary, logistic regression achieved the highest AUC and specificity in the test set. DCA was further performed to assess the clinical utility of the four models (**Figures 6C, D**). In both the training and test sets, the models generally provided greater net benefit than the reference strategies of identifying all participants as PDC or identifying none as PDC across much of the displayed threshold probability range. Random forest and XGBoost showed relatively higher net benefit across parts of the threshold probability range, whereas logistic regression also maintained positive net benefit across clinically relevant thresholds. Overall, dietary preferences provided useful information for PDC identification. Logistic regression showed the best discriminative performance in the test set, whereas DCA indicated that the relative clinical utility of the models varied across threshold probabilities.

**Figure 6:**
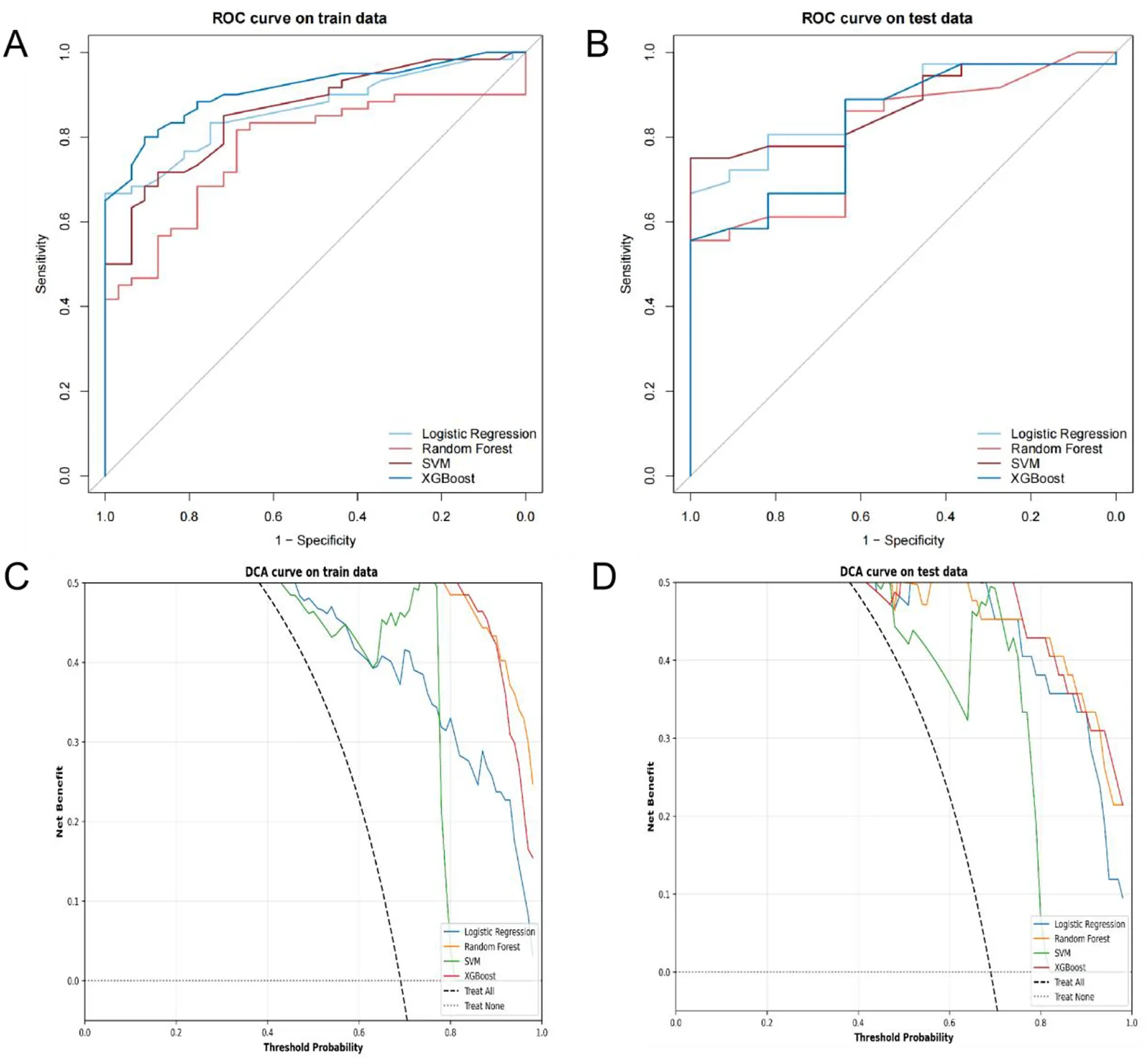
ROC curves and decision curve analysis (DCA) of the dietary-preference models developed using four machine-learning methods in the training and test datasets. **(A)** ROC curve for the training data. **(B)** ROC curve on the testing data. **(C)** Decision curve analysis in the training dataset. **(D)** Decision curve analysis in the test dataset.

A main effect analysis was performed using 10 dietary preferences as feature variables to enhance the understanding of the role of dietary preferences in predicting PDC. **Figure 7A** displayed the SHAP values of these features, illustrating the distribution of color-coded points. This representation underscored the notable dietary preferences of scorching, salty, and light in predicting PDC. Both scorching and salty showed positive and nonlinear associations with PDC, indicating that participants with these preferences were at a higher risk for PDC predictions. However, light dietary preference revealed a distinct negative correlation with PDC, suggesting a lower risk for PDC. This suggested that dietary preferences were significant factors in predicting PDC predictions.

**Figure 7:**
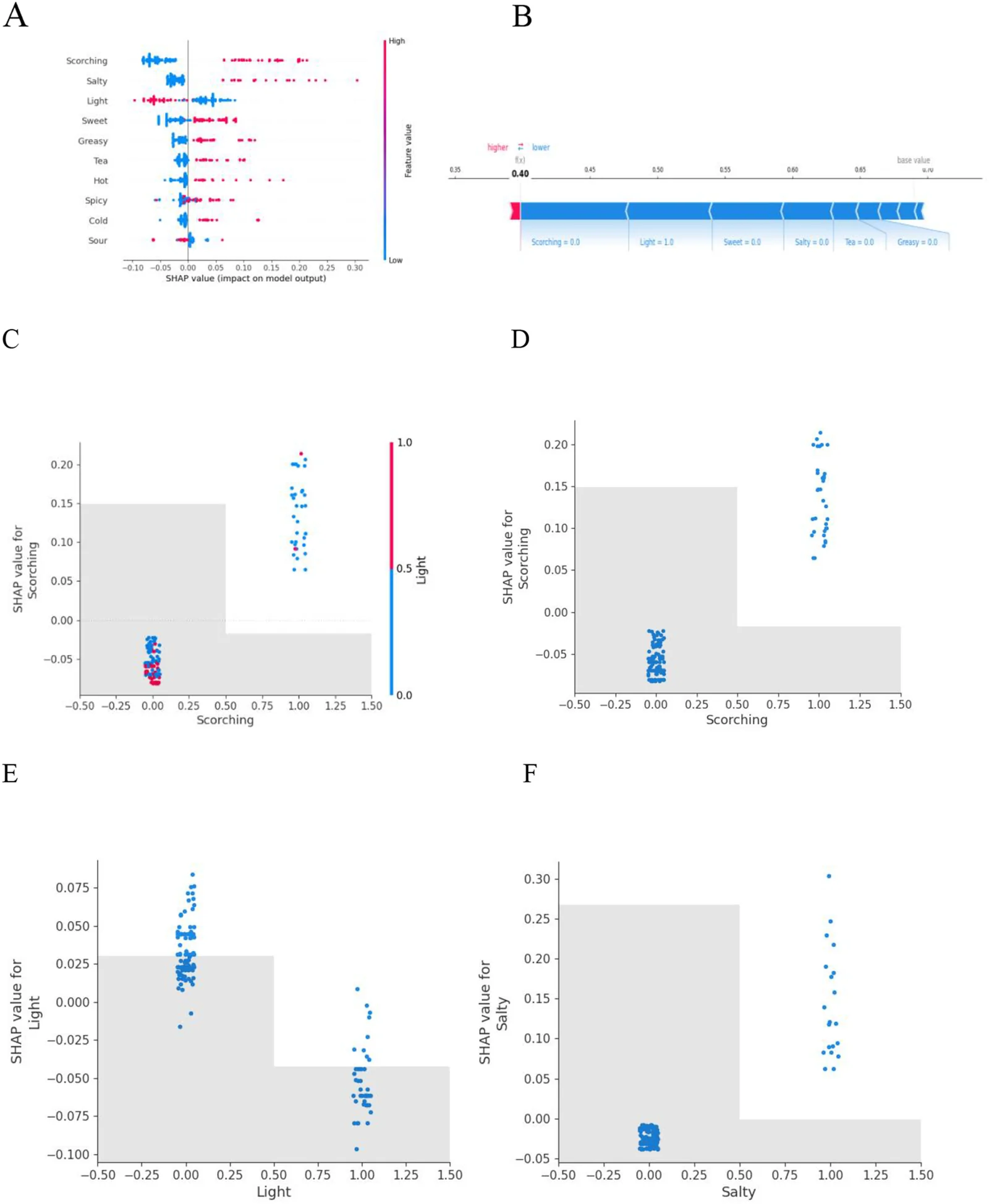
Model interpretation using SHAP. **(A)** SHAP summary plot of feature attributes. **(B)** SHAP force plot of feature attributes. **(C)** SHAP interaction values between scorching and light. **(D)** Dependence plots for SHAP values of scorching. **(E)** Dependence plots for SHAP values of light. **(F)** Dependence plots for SHAP values of salty.

In addition, we provided a representative case to investigate the operational logic of the model. **Figure 7B** displays the SHAP force plot, which highlights the impact of dietary preferences on the model’s prediction of PDC likelihood, in comparison to the baseline. The results indicated a lower predicted probability of PDC for this participant. In the analysis of interactions between scorching and light flavors, participants with a preference for the scorching flavor had higher SHAP values than those with a preference for the light flavor (**Figure 7C**). This indicated that a preference for scorching flavor could significantly predict the risk of PDC. In contrast, the SHAP values for the Light flavor had insignificant influence. Therefore, we infer that a preference for Scorching flavor strongly predicts PDC classification. Further analysis revealed that the values of 0 consistently fell within the negative range, whereas the values of 1 were exclusively within the positive range (**Figures 7D, F**). The data for light in **Figure 7E** indicate a contrasting pattern. These findings indicate that scorching and salty preferences shifted the model output toward PDC, whereas a light preference shifted it toward BC.

## Discussion

Developing a non-invasive PDC prediction model that is accurate, convenient, and easily implementable is essential for addressing the global rise in metabolic diseases (22–24). By combining ML technology with participants’ clinical data and biochemical indicators, the interpretable prediction model developed in this study supports the wider integration of key health concepts, such as the role of TCM constitutional theory in disease prevention, into diverse areas of healthcare practice (25, 26). Recent studies have also highlighted the urgent need for intervention for individuals at high risk of metabolic dysfunction (27, 28). Consequently, the identification of individuals with PDC for early prevention of various metabolic disorders has emerged as a pressing research priority.

PDC is characterized by excessive phlegm and dampness in the body, along with a propensity for obesity, often indicative of metabolic disorder and sluggishness in the body (8, 9). In this paper, we performed statistical analysis and LASSO regression on 17 clinical indicators obtained from 139 enrolled participants. Subsequently, multivariable prediction models (M1-M6) were developed using the identified PDC-associated clinical indicators, including age, BMI, WC, TG, TC, HDLC, LDLC, SBP, DBP, FBG, 2hPG, and UA. The models were applied using ML techniques to predict PDC. Among the tested models, logistic regression M1, which contained WC and TG, achieved a joint-highest AUC of 0.983, with a sensitivity of 0.945, a specificity of 0.970, and a PPV of 0.990, demonstrating strong overall performance. In addition, M1, although it used fewer indicators, demonstrated excellent performance in identifying PDC. This finding suggests that a relatively small set of body-size and lipid-related variables may provide sufficient information for PDC identification in this cohort.

Of note, the features used in M2, including age, BMI, SBP, DBP, and WC, are all non-invasive and can be obtained without blood collection or laboratory testing. Considering that M1 incorporates blood-based indicators (TG), the comparable predictive performance of the two models further confirms its potential as a valuable, non-invasive alternative for identifying PDC. Thus, the non-invasive nature of M2 may facilitate its further evaluation in clinical and community screening settings. Thus, we conclude that M2 performs exceptionally well and offers substantial clinical value for the non-invasive identification of PDC. In routine outpatient visits or community health examinations, readily available information such as age, BMI, waist circumference, and blood pressure could be collected without additional laboratory testing and used for preliminary PDC screening. Individuals identified as having a higher probability of PDC could then undergo further TCM constitution assessment or clinical evaluation.

Furthermore, results of the SHAP analysis demonstrated the contribution of individual features to the prediction outcomes, enhancing the interpretability of ML (29, 30). The SHAP results further indicated that BMI, TG, FBG, and SBP were among the major contributors to model predictions. These variables reflect adiposity, lipid metabolism, glycemic regulation, and blood-pressure status, respectively, suggesting that the model primarily captured anthropometric and metabolic characteristics associated with PDC. From a clinical perspective, the prominence of routinely obtainable variables such as BMI and SBP also supports the potential value of low-burden clinical measurements for PDC identification.

Overall, the selected indicators are closely associated with metabolic diseases, demonstrating a strong association between PDC and metabolic dysfunction (31, 32). Given the prolonged and often insidious onset of metabolic diseases (3), early identification of PDC holds significant clinical value in both prevention and early detection. Research has shown that early detection and management of metabolic dysfunction allows the initiation of appropriate strategies to delay disease progression, which reduces the overall financial burden on participants (33, 34). The developed model aligns with this perspective, indicating that weight, BMI, WC, TG, TC, and FBG are important features associated with metabolic diseases and PDC identification (35, 36). Early identification of PDC is crucial as it allows timely adoption of lifestyle and medical interventions, thereby mitigating disease progression to severe metabolic disorders.

Despite the utilization of prediction models incorporating clinical indicators, we have also developed an interpretable prediction model focused on dietary preferences related to PDC. We propose a novel non-invasive ML-based model for classifying PDC status from dietary preference. Intriguingly, it was discovered that identification based solely on dietary tastes yields commendable results in forecasting PDC. Across the clinical-variable and dietary-preference analysis, logistic regression showed the most consistent performance. In the clinical-variable analysis, logistic regression and SVM achieved the same highest AUC, whereas in the dietary-preference analysis, logistic regression achieved the highest test-set AUC and XGBoost showed a larger decline from training-set to test-set performance. Notably, DCA showed relatively higher net benefit for random forest and XGBoost across parts of the threshold probability range, despite the higher test-set AUC of logistic regression. This difference reflects the complementary information provided by discrimination and decision-analytic performance, as DCA evaluates the clinical consequences of model-guided decisions at specific threshold probabilities rather than discrimination alone. This pattern may be related to the modest sample size and the relatively limited set of structured clinical and dietary predictors, which may have reduced the benefit of more flexible nonlinear models. Under these conditions, simpler logistic regression may provide more stable generalization, whereas more complex algorithms may be more susceptible to overfitting. Moreover, the use of models avoids the need for invasive procedures, such as blood sampling, and does not rely on specialized clinical skills, making it an efficient method for predicting PDC.

Previous studies have demonstrated the feasibility of data-driven TCM constitution identification using questionnaire-derived, genetic, laboratory-based, or imaging features (11–14). These studies established the feasibility of data-driven constitution identification but relied primarily on questionnaire-derived, molecular, laboratory-based, or image-derived information. In the present study, routinely obtained variables, including BMI, WC, SBP, and DBP, provided sufficient discriminatory information to support a fully non-invasive candidate model, while dietary preferences offered an additional low-burden source of information. This complementary design extends previous PDC modeling research by combining routine clinical assessment with self-reported dietary information, neither of which requires specialized imaging or additional laboratory testing. Furthermore, the comparison of four algorithms within the same cohort and the use of SHAP enabled the principal predictors to be interpreted at both the overall and individual levels.

Dietary preferences are a crucial external factor influencing human health and the onset of metabolic diseases, thereby playing a significant role in health and metabolic disease prediction (37–39). These preferences are intricately linked to the formation of PDC. Insights from biogerontology have recognized dietary preferences as one of the three essential pillars of health and survival (40). Consequently, we developed an ML model based on 10 dietary preferences to predict PDC. Following SHAP visualization, the findings indicated that preferences for scorching, salty, and light flavors were particularly influential. These preferences are associated with various physiological responses and metabolic pathways that contribute to disease progression (41, 42). By examining their impact on metabolic dysfunctions, we provide valuable insight into how dietary habits can act as predictors for disease onset. A preference for scorching flavors is associated with heightened inflammation and oxidative stress, which contributes to insulin resistance (43, 44). Similarly, a preference for salty tastes is another critical dietary factor linked to metabolic diseases (45, 46). High sodium intake, often associated with salty foods, can lead to increased blood pressure and fluid retention (47). These dietary profiles also contribute to metabolic issues such as hypertension and dyslipidemia, making them valuable risk predictors for these conditions (48, 49). In contrast, a preference for light flavors, characterized by less intense tastes, tends to correlate with healthier metabolic profiles. Individuals in this group often consume more fruits and vegetables (50, 51) and less intense foods (52). In our SHAP analysis, a preference for light flavors shifted model predictions toward BC rather than PDC. By integrating dietary preferences into predictive models for PDC, we demonstrate that taste inclinations such as scorching, salty, and light can serve as significant predictors. This approach facilitates a more efficient and convenient process for healthcare professionals to identify and forecast PDC, thereby improving the effectiveness and clinical utility of prediction tools. Using ML algorithms, the dietary-preference model analyzed dietary patterns for PDC identification and achieved an AUC above 0.8.

Nonetheless, this study has several limitations. First, the selected indicators primarily focus on metabolic diseases, and therefore, further research is needed to identify further contributing factors. Second, the performance of the prediction models may be constrained by the relatively small sample size and the unequal distribution of PDC and BC participants, which may affect model stability and generalizability. Although stratified sampling was used during dataset partitioning to preserve the relative class distribution in the training and test sets, further validation in larger and more balanced populations is warranted.

In future, it is important to test the performance of the model in other TCM constitution types and evaluate its applicability across diverse geographical and ethnic populations. To validate these findings, prospective multicenter studies with larger sample sizes are needed to assess reproducibility. Additionally, further exploration of the predictive power of ML for other constitutional types through extensive clinical studies is essential. Despite these limitations, this study lays a solid foundation for incorporating constitution-based diagnostics into the early detection of metabolic diseases.

## Conclusion

In summary, this study developed interpretable ML-based models for PDC identification using clinical variables and dietary preferences, including a fully non-invasive candidate model. These findings support the potential of accessible PDC identification to facilitate early risk recognition and preventive strategies for metabolic diseases.

## Data Availability

The raw data supporting the conclusions of this article will be made available by the authors, without undue reservation.

## References

[1] Chew NWS Ng CH Tan DJH Kong G Lin C Chin YH . The global burden of metabolic disease: Data from 2000 to 2019. Cell Metab. (2023) 35:414–28. doi: 10.1016/j.cmet.2023.02.003 36889281

[2] Clemente-Suárez VJ Martín-Rodríguez A Redondo-Flórez L López-Mora C Yáñez-Sepúlveda R Tornero-Aguilera JF . New insights and potential therapeutic interventions in metabolic diseases. Int J Mol Sci. (2023) 24:10672. doi: 10.3390/ijms241310672 37445852 PMC10342188

[3] Agus A Clément K Sokol H . Gut microbiota-derived metabolites as central regulators in metabolic disorders. Gut. (2021) 70:1174–82. doi: 10.1136/gutjnl-2020-323071 33272977 PMC8108286

[4] Zhang X Sun Z Sun W Li Y Gao F Teng F . Bioinformatics analysis and experimental findings reveal the therapeutic actions and targets of Cyathulae Radix against type 2 diabetes mellitus. J Diabetes Res. (2024) 2024:5521114. doi: 10.1155/2024/5521114 39534794 PMC11557179

[5] Zou Y Zhang Y Li M Cao K Song C Zhang Z . Regulation of lipid metabolism by E3 ubiquitin ligases in lipid-associated metabolic diseases. Int J Biol Macromol. (2024) 265:130961. doi: 10.1016/j.ijbiomac.2024.130961 38508558

[6] Cell Metabolism editorial team . Preventing metabolic disease: Part I. Cell Metab. (2024) 36:223. doi: 10.1016/j.cmet.2024.01.010 38325334

[7] Wang Q Zhu Y . Epidemiological investigation of constitutional types of Chinese medicine in general population: Base on 21,948 epidemiological investigation data of nine provinces in China. China J Traditional Chin Med Pharm. (2009) 24:7–12.

[8] Wang Q . Classification and diagnosis basis of nine basic constitutions in Chinese medicine. J Beijing Univ Traditional Chin Med. (2005) 28:1–8.

[9] Shin J Li T Zhu L Wang Q Liang X Li Y . Obese individuals with and without phlegm-dampness constitution show different gut microbial composition associated with risk of metabolic disorders. Front Cell Infect Microbiol. (2022) 12:859708. doi: 10.3389/fcimb.2022.859708 35719350 PMC9199894

[10] Wu X Gu X Huang J Ge N . Analysis of the distribution of TCM constitution and TCM syndrome types in diabetes mellitus and diabetic kidney disease. J Guangzhou Univ Traditional Chin Med. (2023) 40:1863–9. doi: 10.13359/j.cnki.gzxbtcm.2023.08.002

[11] Sun W Bai M Wang J Wang B Liu Y Wang Q . Machine learning-assisted rapid determination for traditional Chinese medicine constitution. Chin Med. (2024) 19:127. doi: 10.1186/s13020-024-00992-0 39278905 PMC11403957

[12] Li J Zhai Y Cao Y Xia Y Yu R . Development of an interpretable machine learning model associated with genetic indicators to identify Yin-deficiency constitution. Chin Med. (2024) 19:71. doi: 10.1186/s13020-024-00941-x 38750482 PMC11094929

[13] Huang Y Guo S Yang J Tang Y Zhu X Ren S . An objective diagnosis model with integrated metabolic and immunity parameters for phlegm-dampness constitution. Evidence-Based Complementary Altern Med. (2022) 2022:3353549. doi: 10.1155/2022/3353549 35154341 PMC8837425

[14] Wang Z Yang T Zhou Z Hu K . Phlegm-dampness and yin-deficiency constitution identification model based on human body 3D reconstruction. J Nanjing Univ Traditional Chin Med. (2024) 40:1340–7. doi: 10.14148/j.issn.1672-0482.2024.1340

[15] Zhang X Xu Q Wen C Luo Y . Construction and optimization of traditional Chinese medicine constitution prediction models based on deep learning. Digital Chin Med. (2024) 7:241–55. doi: 10.1016/j.dcmed.2024.12.00442574925

[16] Peinado BRR Frazão DR Bittencourt LO de Souza-Rodrigues RD Vidigal MTC da Silva DT . Is obesity associated with taste alterations? A systematic review. Front Endocrinol. (2023) 14:1167119. doi: 10.3389/fendo.2023.1167119 37334283 PMC10273260

[17] Zhu Y Wang Q Dai Z Origasa H Di J Wang Y . Case-control study on the associations between lifestyle-behavioral risk factors and phlegm-wetness constitution. J Traditional Chin Med. (2014) 34:286–92. doi: 10.1016/s0254-6272(14)60092-3 24992755

[18] Medicine CAOC . Classification and judgment of traditional Chinese medicine constitution (ZYYXH/T157-2009). World J Integrated Traditional Western Med. (2009) 4:303–4. doi: 10.13935/j.cnki.sjzx.2009.04.015c

[19] Zhu J Gao R Zhao S Lu G Zhao D Li J . Guidelines for the prevention and treatment of dyslipidemia in Chinese adults (2016 revision). Chin Circ J. (2016) 31:937–53. doi: 10.3969/j.issn.1000-3614.2016.10.001

[20] Zhao C Li J Liu J Cao J . Correlation between different blood glucose levels and metabolic syndrome in elderly patients with ischemic stroke. Chin J Gerontology. (2014) 34:4661–2. 32722780

[21] Tang G Qi L Sun Z Liu J Lv Z Chen L . Evaluation and analysis of incidence and risk factors of lower extremity venous thrombosis after urologic surgeries: A prospective two-center cohort study using LASSO-logistic regression. Int J Surg. (2021) 89:105948. doi: 10.1016/j.ijsu.2021.105948 33892158

[22] Su X Cheung CYY Zhong J Ru Y Fong CHY Lee C-H . Ten metabolites-based algorithm predicts the future development of type 2 diabetes in Chinese. J Adv Res. (2024) 64:131–42. doi: 10.1016/j.jare.2023.11.026 38030128 PMC11464468

[23] Shahabi F Battalio SL Pfammatter AF Hedeker D Spring B Alshurafa N . A machine-learned model for predicting weight loss success using weight change features early in treatment. NPJ Digital Med. (2024) 7:344. doi: 10.1038/s41746-024-01299-y 39613928 PMC11607303

[24] Zhang Z Wang S Zhu Z Nie B . Identification of potential feature genes in non-alcoholic fatty liver disease using bioinformatics analysis and machine learning strategies. Comput Biol Med. (2023) 157:106724. doi: 10.1016/j.compbiomed.2023.106724 36898287

[25] Nielsen RL Monfeuga T Kitchen RR Egerod L Leal LG Schreyer ATH . Data-driven identification of predictive risk biomarkers for subgroups of osteoarthritis using interpretable machine learning. Nat Commun. (2024) 15:2817. doi: 10.1038/s41467-024-46663-4 38561399 PMC10985086

[26] Chen L Li F Wang Q Wang J Zhao X . Discussion on proactive health based on the traditional Chinese medicine constitution theory. China J Traditional Chin Med Pharm. (2022) 37:4315–8.

[27] Xu Y Lu J Li M Wang T Wang K Cao Q . Diabetes in China part 2: prevention, challenges, and progress. Lancet Public Health. (2024) 9:e1098–104. doi: 10.1016/s2468-2667(24)00251-2 39579775

[28] Sun Q Yu D Fan J Yu C Guo Y Pei P . Healthy lifestyle and life expectancy at age 30 years in the Chinese population: An observational study. Lancet Public Health. (2022) 7:e994–e1004. doi: 10.1016/s2468-2667(22)00110-4 35926549 PMC7615002

[29] Qi X Wang S Fang C Jia J Lin L Yuan T . Machine learning and SHAP value interpretation for predicting comorbidity of cardiovascular disease and cancer with dietary antioxidants. Redox Biol. (2025) 79:103470. doi: 10.1016/j.redox.2024.103470 39700695 PMC11729017

[30] Bernard D Doumard E Ader I Kemoun P Pagès J Galinier A . Explainable machine learning framework to predict personalized physiological aging. Aging Cell. (2023) 22:e13872. doi: 10.1111/acel.13872 37300327 PMC10410015

[31] Bray GA . Beyond BMI. Nutrients. (2023) 15:2254. doi: 10.3390/nu15102254 37242136 PMC10223432

[32] Ross R Neeland IJ Yamashita S Shai I Seidell J Magni P . Waist circumference as a vital sign in clinical practice: A consensus statement from the IAS and ICCR Working Group on Visceral Obesity. Nat Rev Endocrinol. (2020) 16:177–89. doi: 10.1038/s41574-019-0310-7 32020062 PMC7027970

[33] Wu Q Li J Sun X He D Cheng Z Li J . Multi-stage metabolomics and genetic analyses identified metabolite biomarkers of metabolic syndrome and their genetic determinants. EBioMedicine. (2021) 74:103707. doi: 10.1016/j.ebiom.2021.103707 34801968 PMC8605407

[34] Handelsman Y Butler J Bakris GL DeFronzo RA Fonarow GC Green JB . Early intervention and intensive management of patients with diabetes, cardiorenal, and metabolic diseases. J Diabetes Complications. (2023) 37:108389. doi: 10.1016/j.jdiacomp.2022.108389 36669322

[35] Sanjari M Hadavizadeh M Sadeghi N Naghibzadeh-Tahami A . Effect of empagliflozin on weight in patients with prediabetes and diabetes. Sci Rep. (2025) 15:118. doi: 10.1038/s41598-024-83820-7 39748045 PMC11696924

[36] Aronne LJ Sattar N Horn DB Bays HE Wharton S Lin W-Y . Continued treatment with tirzepatide for maintenance of weight reduction in adults with obesity: The SURMOUNT-4 randomized clinical trial. JAMA. (2024) 331:38–48. doi: 10.1001/jama.2023.24945 38078870 PMC10714284

[37] Hu FB . Diet strategies for promoting healthy aging and longevity: An epidemiological perspective. J Internal Med. (2024) 295:508–31. doi: 10.1111/joim.13728 37867396 PMC10939982

[38] Ventura AK Phelan S Silva Garcia K . Maternal diet during pregnancy and lactation and child food preferences, dietary patterns, and weight outcomes: A review of recent research. Curr Nutr Rep. (2021) 10:413–26. doi: 10.1007/s13668-021-00366-0 34383279

[39] Katsiki N Stoian AP Rizzo M . Dietary patterns in non-alcoholic fatty liver disease (NAFLD): Stay on the straight and narrow path! Clin Invest Arterioscler. (2022) 34:S24–31. doi: 10.1016/j.arteri.2021.10.001 35131122

[40] Rattan SIS Kaur G . Nutrition, food and diet in health and longevity: We eat what we are. Nutrients. (2022) 14:5376. doi: 10.3390/nu14245376 36558535 PMC9785741

[41] Madaris TR Venkatesan M Maity S Stein MC Vishnu N Venkateswaran MK . Limiting Mrs2-dependent mitochondrial Mg^2+^ uptake induces metabolic programming in prolonged dietary stress. Cell Rep. (2023) 42:112155. doi: 10.1016/j.celrep.2023.112155 36857182 PMC10134742

[42] Witkamp RF . Nutrition to optimise human health—How to obtain physiological substantiation? Nutrients. (2021) 13:2155. doi: 10.3390/nu13072155 34201670 PMC8308379

[43] Piątkowska-Chmiel I Krawiec P Ziętara KJ Pawłowski P Samardakiewicz M Pac-Kożuchowska E . The impact of chronic stress related to COVID-19 on eating behaviors and the risk of obesity in children and adolescents. Nutrients. (2023) 16:54. doi: 10.3390/nu16010054 38201884 PMC10780384

[44] González P Lozano P Ros G Solano F . Hyperglycemia and oxidative stress: An integral, updated and critical overview of their metabolic interconnections. Int J Mol Sci. (2023) 24:9352. doi: 10.3390/ijms24119352 PMC10253853 37298303

[45] Li Q Jin R Yu H Lang H Cui Y Xiong S . Enhancement of neural salty preference in obesity. Cell Physiol Biochem. (2017) 43:1987–2000. doi: 10.1159/000484122 29055956

[46] Yang HJ Song MY Kim MJ Park S . Associations between metabolic syndrome and urinary Na-to-K ratio and glomerular filtration rate in middle-aged adults regardless of Na and K intakes. Clin Exp Nephrol. (2020) 24:1015–24. doi: 10.1007/s10157-020-01933-5 32700113

[47] Rust P Ekmekcioglu C . Impact of salt intake on the pathogenesis and treatment of hypertension. Adv Exp Med Biol. (2017) 956:61–84. doi: 10.1007/5584_2016_147 27757935

[48] Mozaffarian D . Dietary and policy priorities for cardiovascular disease, diabetes, and obesity: A comprehensive review. Circulation. (2016) 133:187–225. doi: 10.1161/circulationaha.115.018585 26746178 PMC4814348

[49] Mahmood L González-Gil EM Schwarz P Herrmann S Karaglani E Cardon G . Frequency of family meals and food consumption in families at high risk of type 2 diabetes: The Feel4Diabetes-study. Eur J Pediatr. (2022) 181:2523–34. doi: 10.1007/s00431-022-04445-4 35353229 PMC9110493

[50] Nishi SK Khoury N Valle Hita C Zurbau A Salas-Salvadó J Babio N . Vegetable and fruit intake variety and cardiovascular health and mortality: A systematic review and meta-analysis of observational studies. Nutrients. (2023) 15:4913. doi: 10.3390/nu15234913 38068771 PMC10707746

[51] Madsen H Sen A Aune D . Fruit and vegetable consumption and the risk of hypertension: A systematic review and meta-analysis of prospective studies. Eur J Nutr. (2023) 62:1941–55. doi: 10.1007/s00394-023-03145-5 37106252 PMC10349693

[52] Khanna SK . Understanding the impact of globalization on food preferences, dietary patterns, and health. Ecol Food Nutr. (2016) 55:339–40. doi: 10.1080/03670244.2016.1213557 27454623

