# Supplementary material for: An interpretable prediction model for non-invasive identification of the phlegm-dampness constitution based on machine learning
Source: Front Endocrinol (Lausanne). 2026 Sep 22;17:1929926. doi: 10.3389/fendo.2026.1929926 (PMC13638588; doi:10.3389/fendo.2026.1929926)
Supplement: Supplementary file 1 [file Table1.docx]

**Table S1. Hyperparameter search ranges and selected values for M1–M6.**

| **Algorithm** | **Parameter** | **Search range** | **Selected values for M1–M6** |
| --- | --- | --- | --- |
| LR | C | 0.001–1000 | 10, 0.316, 1, 3.162, 0.316, 1 |
| LR | penalty | l1, l2 | l2, l2, l2, l1, l2, l2 |
| LR | max_iter | 500–2000 | 500, 500, 500, 500, 500, 500 |
| RF | n_estimators | 50–200 | 100, 50, 50, 100, 50, 100 |
| RF | max_depth | 3–8 | 5, 5, 3, 3, 5, 3 |
| RF | min_samples_split | 5, 10 | 5, 5, 5, 5, 5, 5 |
| RF | min_samples_leaf | 5, 10 | 5, 5, 10, 5, 5, 5 |
| SVM | kernel | linear, rbf | linear for M1–M6 |
| SVM | C | 0.1–10 | 10, 0.1, 10, 1, 0.1, 10 |
| SVM | gamma | scale, 0.01 | scale for M1–M6 |
| XGBoost | max_depth | 2–4 | 2, 2, 2, 3, 2, 3 |
| XGBoost | learning_rate | 0.01, 0.1 | 0.01, 0.01, 0.1, 0.1, 0.01, 0.01 |
| XGBoost | subsample | 0.6, 0.8 | 0.8, 0.8, 0.8, 0.6, 0.8, 0.6 |
| XGBoost | colsample_bytree | 0.6, 0.8 | 0.6, 0.6, 0.8, 0.8, 0.6, 0.6 |
| XGBoost | reg_lambda | 1, 5 | 1, 1, 1, 1, 1, 1 |

M1: “WC+TG”, M2: “SBP+DBP+WC”, M3: “TC+TG+HDLC+LDLC+FBG+2hPG+UA”, M4: “SBP+DBP+WC+TC+TG+HDLC+LDLC+
FBG+2hPG+UA”, M5: “WC”, M6: “TG”, and age and BMI covariates were added to M1-M6.

Note: Selected values are listed in the order M1, M2, M3, M4, M5, and M6.

Additional fixed settings: RF max_features = sqrt and class_weight = balanced; SVM class_weight = balanced; XGBoost scale_pos_weight = 0.448.
